# Predicting the Freshness of Starch-Coated Snakehead Fish Fillets During Storage Using Hyperspectral Imaging Combined with Transfer Learning

**DOI:** 10.3390/foods15122191

**Published:** 2026-06-17

**Authors:** Mingyuan Sha, Zemao Chen, Jingxiao Yu, Qingyi Wei

**Affiliations:** 1 Jinan University-University of Birmingham Joint Institute, Jinan University, Guangzhou 511443, China; 2 School of Food Science and Engineering, South China University of Technology, Guangzhou 510641, China; 3 Academy of Contemporary Food Engineering, South China University of Technology, Guangzhou Higher Education Mega Center, Guangzhou 510006, China

**Keywords:** hyperspectral imaging, transfer learning, starch-coated snakehead fish fillet, freshness prediction

## Abstract

Freshness prediction of starch-coated snakehead fish fillets across different storage times remains challenging due to complex quality deterioration and spectral distribution shifts. In the current research, hyperspectral imaging (HSI) combined with transfer learning (TL) was developed to predict the freshness of starch-coated snakehead fish fillets during short-term refrigerated and long-term frozen storage. The results showed that storage led to texture deterioration, pH increase, TVB-N accumulation, and lipid oxidation, while starch coating effectively delayed quality degradation. Compared with models based only on short-term or long-term data, the domain transfer convolutional neural network (DT-CNN) model improved the robustness of freshness prediction across storage stages. The DT-CNN model based on VIS spectra achieved the best performance for TBA prediction in the starch coating treatment group, with an *R*_P_^2^ of 0.76 and RMSE_P_ of 0.13, and showed strong performance for TVB-N prediction in the starch coating treatment group, with an *R*_P_^2^ of 0.85 and RMSE_P_ of 8.66. These results demonstrate that HSI combined with TL is a promising non-destructive method for freshness evaluation of starch-coated snakehead fish fillets during storage.

## 1. Introduction

Fish products are highly susceptible to a series of changes during storage, including protein degradation, lipid oxidation, water migration, and deterioration of tissue structure [1,2]. Furthermore, the above changes can lead to a decline in flavor, poor texture, and a shortened shelf life [3]. For processed aquatic products with high protein and water content, such as snakehead fish fillets, the storage phase not only directly determines the final edible quality of the product but also affects the subsequent distribution stability and market acceptance [4]. Published research indicates that fish products exhibit distinct patterns of quality deterioration under different storage conditions, and snakehead fish fillets also undergo significant quality changes when stored under frozen or refrigerated conditions [5]. Therefore, a systematic study of the quality changes in snakehead fish fillets during storage will not only assist in elucidating their deterioration characteristics but also provide a theoretical basis for process optimization and quality control.

Starch-coated snakehead fish fillets are a key product category in the current deep processing of snakehead fish [6]. Starch-coated treatment essentially refers to improving the surface condition and internal water-holding behavior of fish fillets through batter conditioning, thereby enhancing the tenderness, smoothness, stability, and cooking adaptability [7]. Generally, starch-coated treatment can mitigate issues such as water loss and coarse texture during heating to some extent. However, starch-coated treatment may also alter the quality evolution pathway of the fillets during storage [8]. For example, the batter system may influence the surface barrier properties, internal water-binding state, and oxidative processes of the samples, thereby altering the texture retention capacity and freshness dynamics of the fillets during storage [9,10]. Previous studies have shown that different pretreatment methods significantly affect the quality evolution and processing suitability of snakehead fish fillets [11]. Therefore, it is necessary to systematically analyze the impact of starch-coated treatment on the changes in the storage quality of snakehead fish fillets by comparing the control group with the starch-coated treatment group.

Currently, the evaluation of fish storage quality and freshness is primarily based on physicochemical indicators such as texture, total volatile basic nitrogen (TVB-N) and thiobarbituricacid (TBA). Among these, TVB-N can reflect the extent of protein degradation and the accumulation of volatile basic nitrogen compounds [12]. TBA is used to characterize the level of lipid oxidation [13]. However, these traditional detection methods typically require sampling, homogenization, and chemical analysis. In addition, the testing process is destructive and fails to meet the practical needs of rapid screening. In recent years, hyperspectral imaging (HSI) technology has demonstrated significant application potential in freshness identification, monitoring of storage quality changes, and non-destructive evaluation of fish fillets due to its ability to simultaneously acquire spatial and continuous spectral information [14,15]. Furthermore, HSI technology has been employed to characterize the microbial, chemical, and biochemical changes occurring in fish fillets during storage, yielding favorable results in the non-destructive evaluation of textural properties [16,17]. However, research on the changes in storage quality of starch-coated snakehead fish fillets and the corresponding spectral response patterns remains relatively limited.

With the development of artificial intelligence (AI), the convolutional neural network (CNN) has been increasingly applied to hyperspectral data analysis due to the ability to automatically extract nonlinear features from high-dimensional spectral information [18]. Compared with traditional chemometric methods, CNN models can reduce the dependence on manual feature extraction and improve the representation of complex relationships. For example, Che et al. [19] used HSI combined with a CNN model to predict texture profile analysis parameters of live spotted seabass and achieved effective visualization of muscle texture distribution. In addition, Kai et al. [20] applied HSI combined with a 1D-CNN model to predict the moisture content of bighead carp fillets during microwave drying, demonstrating the potential of HSI based on CNN for fish quality evaluation. However, most existing studies on fish quality assessment have focused on model development within a single storage condition or a single data domain. This phenomenon may limit the generalization ability and practical applicability of the developed models when they are applied to samples under different storage conditions or data distributions.

In the food analysis field, transfer learning (TL) has been regarded as an effective strategy for reducing the dependence on large-scale labeled samples by transferring generalizable spectral features from a data-rich source domain to a data-limited target domain [21]. In practical research and applications, long-term frozen storage experiments generally require extended storage periods, continuous sampling, and repeated physicochemical measurements, resulting in high experimental costs and limited sample availability [22]. In contrast, short-term refrigerated storage samples are easier to obtain and can provide relatively sufficient spectral and quality-related data. Although short-term refrigerated storage and long-term frozen storage differ in storage temperature, deterioration rate, and the degree of quality degradation, both processes involve the quality evolution of snakehead fish fillets, which is closely associated with protein degradation, lipid oxidation, water migration, and structural changes [23,24]. It should be emphasized that the short-term refrigerated samples were not used as a substitute for long-term frozen storage samples. Instead, they were used as auxiliary source-domain data in the TL framework, while the long-term frozen storage samples were used as the target domain for model adaptation and evaluation. Therefore, the two storage domains may still share certain common response patterns between spectra and quality data. From the perspective of TL [25], short-term refrigerated storage samples can be regarded as the source domain, while long-term frozen storage samples can be regarded as the target domain. By learning generalizable spectral features and quality-related representations from the data-rich source domain and transferring them to the data-limited target domain, the model can reduce the dependence on a large number of long-term storage samples [26]. Meanwhile, appropriate domain adaptation or fine-tuning can help alleviate the distribution differences between refrigerated and frozen storage conditions. Therefore, utilizing the effective spectral information contained in short-term storage samples to assist long-term storage prediction is expected to overcome the modeling limitations caused by insufficient target-domain samples and improve the adaptability, generalization ability, and predictive stability of models for long-term frozen storage samples.

Therefore, this study aimed to develop a framework combining HSI with TL for the rapid freshness assessment of starch-coated snakehead fish fillets during storage. The changes in texture, pH, TVB-N, and TBA were analyzed to characterize quality deterioration, while VIS and NIR spectral information was used to establish predictive models for indicators related to freshness. In particular, short-term storage samples were used to assist in establishing models for long-term storage samples through the TL method, thereby addressing the challenge of limited long-term storage data. This study provides a potential technical foundation for freshness monitoring and rapid quality assessment of starch-coated fish fillets.

## 2. Materials and Methods

### 2.1. Overall Experimental Design

**Figure 1:**
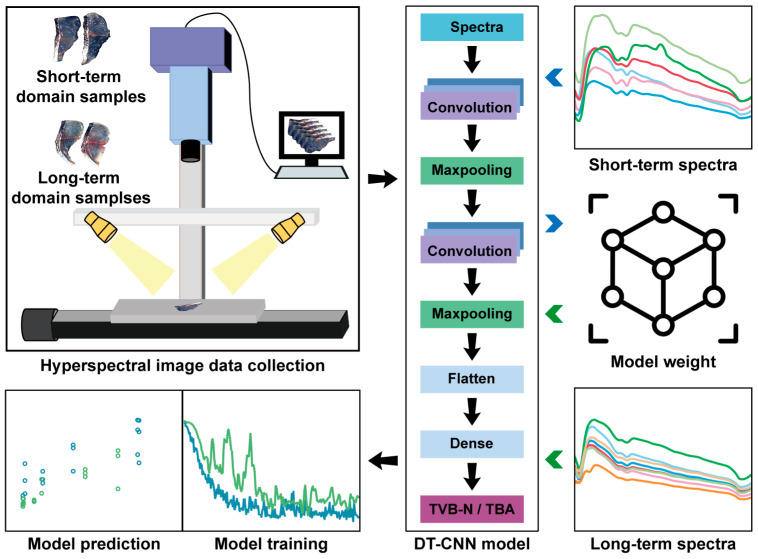
Schematic diagram illustrating the freshness prediction of starch-coated snakehead fish fillets using HSI technology combined with the DT-CNN model. The black arrows indicate the overall workflow. The blue arrows represent the source-domain information derived from short-term spectra, whereas the green arrows represent the target-domain modeling process using long-term spectra and transferred model weights for TVB-N and TBA prediction.

The overall experimental workflow of this study is shown in Figure 1. Snakehead fish fillets from the control group and starch-coating treatment group were used as research samples. During storage, texture, pH, TVB-N, and TBA were measured to evaluate freshness changes and quality deterioration. Meanwhile, VIS and NIR hyperspectral images were collected at different storage times, and the corresponding spectral information was extracted for model development. Then, a domain transfer convolutional neural network (DT-CNN) model was constructed to predict TVB-N and TBA values. In the TL strategy, short-term storage samples were used as the source domain, whereas long-term storage samples were used as the target domain. Furthermore, the model performance was evaluated to investigate the feasibility of TL for freshness prediction under limited long-term storage samples.

### 2.2. Preparation of Samples 

The snakehead fish samples were sourced from a commercial aquaculture facility (Foshan, China). The samples were fish fillets obtained after dissection. The fillets were cut into slices approximately 2 mm thick, which were divided into a control group (fish slices without starch coating) and a treatment group (fish slices with starch coating). The slice thickness was obtained using a mechanical slicer with a nominal blade interval of 2 mm to standardize sample geometry for starch coating, hyperspectral image acquisition, and physicochemical measurements. The treatment group used a tumbling method. During the tumbling process, potato starch equivalent to 10% of the fish fillet weight and an equal amount of water were added to ensure uniform coating of the fish fillet surface, thereby obtaining starch-coated snakehead fish fillets.

After sample preparation, samples from both groups were placed in self-sealing plastic bags and stored at 4 °C for short-term refrigerated storage and at −18 °C for long-term frozen storage, respectively. For short-term storage, texture parameters, pH value, TVB-N content, TBA content, and hyperspectral images were collected at 0, 3, 6, 9, 12, and 15 days. For long-term storage, TVB-N content, TBA content, and hyperspectral images were collected monthly at 0, 1, 2, 3, 4, 5, 6, and 7 months.

### 2.3. Quality Detection

#### 2.3.1. Texture Information

The texture profile analysis (TPA) mode of a texture analyzer (TA.XTplusC, Stable Micro Systems Ltd., London, UK) was used to measure texture properties with the P50 probe [12]. The test conditions are as follows: the pre-test speed was 1.0 mm/s, test speed was 1.0 mm/s, post-test speed was 5.0 mm/s, compression ratio was 50%, interval time was 5 s, and compression distance was 5 mm. The test was conducted using automatic triggering.

#### 2.3.2. pH Value

A pH meter (Model FE20, Mettler-Toledo Co., Zurich, Switzerland) was used to measure the pH value [27]. First, 20 mL of deionized water was added to 2.0 g of sample, and the mixture was homogenized for 30 s at 8000 r/min. Next, the calibrated pH electrode was inserted into the homogenized sample solution for measurement, and the pH value was recorded once the reading stabilized.

#### 2.3.3. TVB-N Content

The automatic Kjeldahl method was used to determine the TVB-N content in samples [28]. First, 10.00 g of sample and 100 mL of ultrapure water were weighed into a conical flask, and the mixture was shaken for 30 min at 25 °C. Then, the mixture was transferred to a distillation tube, and 1 g of magnesium oxide was added for distillation. In addition, 30 mL of boric acid solution and 3 drops of a mixed indicator were added to the receiving flask in advance, and the volume ratio of methyl red to bromophenol blue in the mixed indicator was 1:5. After distillation, titration was performed using a 0.1 mol/L standard hydrochloric acid solution, and the volume of hydrochloric acid consumed was recorded. A reagent blank was determined simultaneously using distilled water in place of the sample. The formula for calculating the TVB-N content in the sample was as follows:

(1) $$\text{TVB-N}=\frac{\left(V_{1}\;-{\;V}_{2}\right)\;\times \;c\;\times \;14\;\times \;100}{m}$$

where TVB-N represents the total volatile basic nitrogen content in the sample (mg/100 g), $V_{1}$ represents the volume of hydrochloric acid standard solution consumed during the sample analysis (mL), $V_{2}$ represents the volume of hydrochloric acid standard solution consumed during the reagent blank analysis (mL), c represents the concentration of the hydrochloric acid standard solution (mol/L), and m represents the mass of the sample (g).

#### 2.3.4. TBA Content

The method for determining TBA content was as follows [29]: 2.000 g of sample and 20 mL of 10% (*w*/*v*) trichloroacetic acid solution were added to a 50 mL round-bottom centrifuge tube and homogenized for 2 min. Subsequently, the mixture was centrifuged at 4 °C and 5100 r/min for 20 min, and 5 mL of the supernatant was collected. Next, 5 mL of 0.02 mol/L thiobarbituric acid solution was added, and the mixture was heated in a 95 °C water bath for 20 min. With ultrapure water as the blank, the absorbance at 532 nm was measured, and the results are expressed in mg MDA/kg.

### 2.4. Hyperspectral Data Acquisition

#### 2.4.1. Hyperspectral Imaging System

Hyperspectral image data of the samples was acquired using the HSI system. This system consists of two data acquisition systems: one for the visible (VIS) band (400–1000 nm) and one for the near-infrared (NIR) band (1000–2500 nm). The VIS spectra data acquisition system primarily includes [30]: a CCD camera (DL-604M, Andor Technology Ltd., Belfast, UK), a spectrometer (Imspector V10E, Spectral Imaging Ltd., Oulu, Finland), a lens (OLE23, Schneider Ltd., Bad Kreuznach, Germany), and a 150 W halogen light source positioned at a 45° to the moving conveyor belt. The NIR spectra data acquisition system primarily consists of [31]: a CCD camera (XC403, Xenics Infrared Solutions, Leuven, Belgium), a spectrometer (Specim V25E, Spectral Imaging Ltd., Oulu, Finland), a lens (OLES30, Xenics Infrared Solutions, Leuven, Belgium), and an illumination unit consisting of two 150 W tungsten-halogen lamps (2900-ER, Illumination Technologies Inc., New York, NY, USA). The common components of the two systems include a translation stage (IRCP0076-1COMB, Isuzu Optics Corp., Taiwan, China) and a computer equipped with data acquisition software (Spectral Image software, Isuzu Optics Corp., Taiwan, China). The data acquisition software can be used to control the wavelength range, motor speed, exposure time, and the image acquisition process.

#### 2.4.2. Data Acquisition and Calibration

The sample was placed on a black background experiment table and secured in a plastic box. During acquisition, the distance between the camera and the sample surface was set to 30 cm, the internal motor speed of the camera was set to 1.1 mm/s, and the exposure time was set to 20 ms. Under the above conditions, reflected hyperspectral images were acquired. In this study, the spatial information collected from hyperspectral images was mainly used to locate the fish fillet region, excluding background areas. Due to factors such as dark current and environmental noise in the HSI system, the raw hyperspectral images typically suffer from a certain degree of systematic error. To minimize the impact of these factors on image quality and subsequent analysis results, black-and-white correction must be applied to the raw images. The method for correcting hyperspectral images was as follows [32]:

(2) $$R=\frac{I-B}{W-B}$$

where R refers to the calibrated hyperspectral image data, I refers to the raw hyperspectral image data, B refers to the hyperspectral image data of a blackboard, and W refers to the hyperspectral image data of a whiteboard. The corrected hyperspectral images were imported into ENVI 5.1 software, and the average reflectance spectra were extracted from the regions of interest.

### 2.5. Data Preprocessing

#### 2.5.1. Data Division

In this study, a total of 240 hyperspectral data points were collected, including 120 samples from the control group and 120 samples from the treatment group. The detailed distribution of samples at each storage time is presented in Table 1. The data were split using the Kennard–Stone (KS) algorithm [33], with 3/4 samples of the dataset allocated to the training set for model development and the remaining 1/4 samples allocated to the testing set for model evaluation. Furthermore, the training set was divided into training and validation data in a 4:1 ratio. Additionally, a 10-fold cross-validation method was employed to repeatedly build models, thereby minimizing the impact of variations and improving model stability.

#### 2.5.2. Spectral Preprocessing

During the acquisition of HSI spectra, various external interferences such as environmental noise, instrumental noise, sample surface unevenness, sample inhomogeneity, and instrumental drift can introduce significant artefacts into the data. These interferences often manifest as spectral noise, light scattering, and baseline drift, compromising the quality of the HSI spectra. To mitigate these issues and enhance the signal-to-noise ratio of the HSI spectral data, spectral preprocessing methods were used to optimize the data. Furthermore, prediction models based on the first-order derivative (1-st der) outperform those using raw spectra and other spectral preprocessing methods. The 1-st der can amplify detailed information and minimize baseline drift effects by calculating the rate of change at each frequency [34]. Therefore, 1-st der will be uniformly applied throughout the current research.

### 2.6. Freshness Prediction Models

#### 2.6.1. DT-CNN Model

The DT-CNN model is a one-dimensional (1D) CNN model that combines spectral feature extraction with domain TL, enhancing the prediction performance and generalization ability of the CNN model under different storage domains. The DT-CNN model sequentially contains one input layer, one convolutional layer (kernel number: 16, kernel size: 9), one maxpooling layer (pooling size: 2, pooling stride: 1), one convolutional layer (kernel number: 32, kernel size: 7), one maxpooling layer (pooling size: 2, pooling stride: 1), one flattened layer, one dense layer (neurons number: 64) and one output layer. The input layer consists of spectral variables, and the output layer consists of 1 neuron referring to TVB-N or TBA. Each convolutional layer was followed by a ReLU activation function, which can enhance the nonlinear fitting ability of the DT-CNN model. The maxpooling layers were used to reduce feature dimensionality and retain the main spectral features. In this study, the model was trained using Adam with a learning rate of 0.0001 and a batch size of 8. For the model training process, the number of training epochs in the short-term domain was 120, and the number of training epochs in the long-term domain was 250 with an early stopping strategy. The RMSE was used as the loss function, and the model performance was evaluated using *R*^2^ and RMSE.

For short-term or long-term fish fillet freshness prediction, this model can be used directly. For fish fillet freshness prediction using TL, the model is first pre-trained on the short-term storage domain and then fine-tuned on the long-term storage domain to achieve domain-transfer prediction. Specifically, the short-term storage samples were used as the source domain to learn general freshness-related spectral features, while the long-term storage samples were used as the target domain for model adaptation and evaluation. During the TL process, the pre-trained model parameters were used to initialize the target-domain model, and all layers were further fine-tuned using the long-term storage data. This strategy was adopted to improve the prediction stability of the model under limited long-term storage samples.

#### 2.6.2. Model Evaluation

In the current research, the coefficient of determination (*R*^2^) and root mean square error (RMSE) were used to evaluate the model performance, which were calculated as follows [35]:

(3) $$R^{2}\;=\;1-\frac{\sum_{i\;=\;1}^{n}{\left(y_{i}\;-\;\hat{y_{i}}\right)}^{2}}{\sum_{i\;=\;1}^{n}{\left(y_{i}\;-\;\bar{y}\right)}^{2}}$$

(4) $$\mathrm{RMSE}=\sqrt{\frac{1}{n}\sum_{i=1}^{n}{\left(y_{i}-\hat{y_{i}}\right)}^{2}}$$

where $R^{2}$ refers to the r-square, n refers to the number of spectral frequency points, i refers to the ith frequency point (i = 1, 2, 3, …, n), $y_{i}$ refers to the transmittance of the ith frequency point of the original spectrum, $\hat{y_{i}}$ refers to the transmittance of the ith frequency point of the fitted spectrum, $\bar{y}$ refers to the mean value of the transmittance of the original spectrum and RMSE refers to the root mean square error.

### 2.7. Statistical Analysis

Measurements of texture information, pH value, TVB-N content, and TBA content were repeated six times and are expressed as mean ± standard deviations. The data were processed for one-way analysis of variance (ANOVA) and the Waller–Duncan post hoc test using the IBM software SPSS Statistics for Windows (17.0, IBM Co., Ltd., Shanghai, China). In addition, the data processing methods including data division, spectral preprocessing, and the building and evaluation of models were based on the Scikit-learn (https://scikit-learn.org/stable/ accessed on 6 May 2026) and Keras (https://keras.io/ accessed on 6 May 2026) frameworks of python 3.10, implemented on the platforms of the Jupyter notebook (https://jupyter.org/ accessed on 6 May 2026) and spyder (https://www.spyder-ide.org/ accessed on 6 May 2026). The figures used to present the research results were drawn using Origin 2021 software (https://www.originlab.com/ accessed on 6 May 2026).

## 3. Results

### 3.1. Texture and pH Analysis

As shown in Table 2, the texture information and pH values of snakehead fish fillets in both the control and treatment groups underwent varying degrees of change during refrigerated storage. In terms of hardness, the values for both groups generally decreased as storage time increased. Specifically, the control group decreased from 4420 g at 0 days to 2546 g at 15 days, a decrease of approximately 42.4%. The treatment group decreased from 3131 g to 1742 g, a decrease of approximately 44.4%. The results indicate that cold storage continuously weakens the ability of tissues to resist deformation under external forces. At the same time, the hardness of the treatment group was generally lower than that of the control group at all storage periods, suggesting that the starch coating treatment can improve tenderness, but it also makes the initial tissue structure softer. Regarding elasticity, the control group decreased from 0.154 to 0.152, showing a relatively small overall change, but a significant decline occurred during days 3 and 6. The treatment group had an initial elasticity of 0.199, then gradually decreased to 0.165. These results indicate that the starch coating treatment can help improve the initial recovery from deformation, but this phenomenon gradually diminishes as storage time increases. Research by Salman et al. [5] indicates that fish products often experience a decrease in hardness and a reduction in texture stability during storage, which is consistent with the pattern of continuously decreasing hardness observed in both groups of samples in this study. Research by Xia et al. [11] indicates that different texture treatments can influence the texture formation process of snakehead fish fillets. Therefore, the phenomenon of the treatment group exhibiting lower initial hardness and higher initial elasticity is reasonable.

In terms of changes in cohesiveness, adhesiveness, and chewiness, the two groups of samples exhibited more complex dynamic characteristics. In the control group, cohesiveness gradually increased from 0.563 to 0.692, while the treatment group decreased from 0.702 to 0.547 before rebounding to 0.664. The results suggest that the samples softened during the later stages of storage, but the degree of inter-tissue bonding may have partially recovered due to water redistribution and matrix rearrangement. Adhesiveness and chewiness better reflect changes in the overall edible quality of the samples. In the control group, adhesiveness decreased from 2497 g to 1826 g between days 0 and 9, with a slight recovery in the later stages. Chewiness decreased from 390 g to 287 g before recovering slightly. The changes in the treatment group were more pronounced. Adhesiveness rapidly decreased from 2201 g to 985 g, then rebounded to 1578 g, while chewiness resistance decreased from 425 g to 192 g before rebounding to 289 g. The above results indicate that samples treated with the starch coating method were more prone to a decline in structural support and a weakening of masticatory properties during the early storage period, but the changes tended to level off later on. The above phenomenon may be related to the starch system formed by the batter absorbing water and swelling during the initial storage period, followed by subsequent structural reorganization. Research by Ramírez et al. [9] indicates that hydrocolloid systems can significantly alter the mechanical properties and functional characteristics of fish products, causing them to exhibit textural evolution patterns during storage that differ from those of untreated samples. Research by Xavier et al. [10] indicates that battering or coating systems exert a persistent influence on the physical quality of fish products, which also provides a rationale for the more pronounced changes in adhesiveness and chewiness observed in the treatment group in this study.

Resilience can reflect the sample’s ability to return to its original shape after being compressed. For both groups, resilience decreased overall as storage time increased. In the control group, the value decreased from 0.303 to 0.194, a decrease of approximately 36.0%. In the treatment group, resilience decreased from 0.343 to 0.215, a decrease of approximately 37.3%. These results indicate that the compression recovery ability of both untreated and starch-coated snakehead fish fillets weakened during refrigerated storage. A previous study [36] reported that storage-induced changes in fish muscle proteins and tissue structure may contribute to textural deterioration. In contrast, the treatment group exhibited higher resilience at 0 days compared to the control group, indicating that the starch-coating treatment may help improve the initial compression recovery ability of the samples. However, the difference between the two groups gradually narrowed as storage time increased, suggesting that the beneficial effect of starch coating treatment on texture retention is primarily evident during the early stages of storage. Overall, refrigerated storage leads to continuous softening and structural deterioration of snakehead fish fillets. Research by Wang et al. [12] indicates that textural parameters can sensitively reflect changes in the quality status and tissue structure of fish products during storage. Studies by Cao et al. [17] demonstrate that parameters such as hardness, chewiness, and resilience are of significant importance in the rapid evaluation of fish meat quality.

During storage, the pH of snakehead fish fillets in both groups initially fluctuated slightly before showing a marked upward trend. The pH of the control group decreased from 6.26 at day 0 to 6.17 at day 3, then gradually increased to 6.51 at day 9. After 12 days, the pH began to rise rapidly, reaching 7.29 on day 15. The initial pH value of the treatment group was 6.33, slightly higher than that of the control group. This pH value dropped to a low of 6.21 on Day 6, then continued to rise, reaching 6.92 by Day 15. Overall, the pH values of both groups changed relatively slowly during the early storage period, but both showed a significant increase after day 9. The above results indicate that the acid–base balance within the samples gradually changed, and spoilage-related metabolites began to accumulate as storage progressed. At the same time, the control group showed a greater increase between days 12 and 15, indicating a more pronounced change in pH during the later stage of storage, while the treatment group showed a slower rise in pH during the same period. This difference suggests that the acid–base status of the two groups changed differently during storage. Considering that pH may be influenced by the sample matrix and starch coating, the pH results were interpreted together with TVB-N and TBA values to more comprehensively evaluate freshness deterioration. Research by Shi et al. [14] indicates that during storage, the pH of fish fillets typically undergoes slight fluctuations in the early stages, followed by a gradual increase as the spoilage process progresses.

### 3.2. TVB-N and TBA Analysis

**Figure 2:**
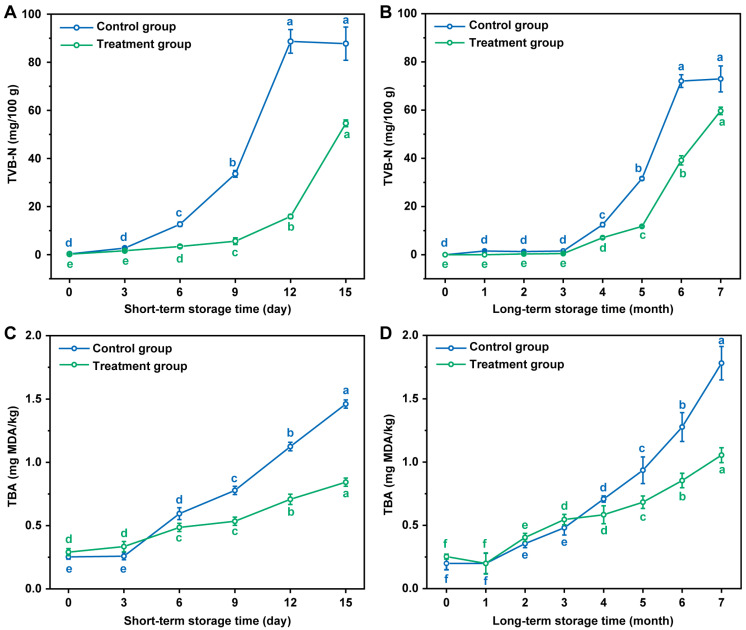
Changes in TVB-N content of control group and treatment group during (**A**) short-term and (**B**) long-term storage stages. Changes in TBA content of the control group and treatment group during (**C**) short-term and (**D**) long-term storage stages. Values are means of three replications ± standard deviation; different lower-case letters in the same row indicate significant difference (*p* < 0.05).

The changes in TVB-N and TBA content are shown in Figure 2. During storage, the TVB-N content of snakehead fish fillets continued to rise with time, but there were significant differences in the rate of change among the groups. During the short-term refrigeration phase (Figure 2A), the TVB-N value in the control group rose slowly between days 0 and 6, but the rate of increase accelerated significantly after day 9, reaching 88.67 mg/100 g by day 12. In contrast, the treatment group showed only a gradual increase between day 0 and 9. Although the increase became pronounced after day 12, the overall level remained lower than that of the control group. The results indicate that as refrigeration time increased, both groups of samples experienced varying degrees of freshness loss. However, the starch coating treatment was able to delay protein degradation and the occurrence of spoilage-related metabolism to some extent, and this effect was more pronounced during the middle and late stages of storage. During the long-term frozen storage phase (Figure 2B), TVB-N values for both groups remained generally low with minimal variation between month 0 and 3 months, indicating that freezing conditions effectively suppressed the accumulation of volatile nitrogen-containing compounds during the early storage period. After 4 months, TVB-N values in both groups began to rise significantly. The control group showed a larger increase between 4 and 6 months, reaching 72.05 mg/100 g at 6 months and continuing to rise at 7 months. In contrast, the treatment group exhibited a relatively gradual increase during the same period. Although a noticeable rise also occurred between 6 and 7 months, the overall values remained lower than those of the control group. These results indicate that there is a certain degree of lag in the quality deterioration of the samples under long-term storage conditions. However, as storage time continues to extend, protein degradation and spoilage metabolism will still gradually accumulate, while the starch coating treatment can continue to play a positive role in delaying this process. This phenomenon may be related to the physical barrier effect of the starch coating layer, which could reduce direct exposure of the fish tissue to oxygen and slow moisture migration, thereby limiting the accumulation of volatile nitrogen-containing compounds to some extent. Similar preservation effects of edible coatings have also been reported in a previous study [37], where coating treatments were found to slow quality deterioration by modifying the surface microenvironment of aquatic products. Nevertheless, the continuous increase in TVB-N in both groups indicates that starch coating could not completely prevent protein degradation and spoilage-related changes during storage.

During both short-term refrigerated and long-term frozen storage, the TBA values of snakehead fish fillets in both groups generally showed an upward trend, indicating that lipid oxidation in the samples progressively increased as storage time extended. For short-term storage (Figure 2C), the initial TBA values of both groups were at relatively low levels, with little change observed between days 0 and 3. Starting from day 6, the TBA value of the control group began to rise significantly higher than that of the treatment group, and the difference gradually widened. By day 15, the control group had risen to a high level. Although the TBA value in the batter-free group continued to increase, the overall rate of increase was significantly lower. These results indicate that both groups of samples underwent continuous oxidative deterioration during refrigeration, but the starch coating treatment exhibited a better inhibitory effect in the middle and late stages of storage, which can help to slow the accumulation of lipid oxidation products. Xiao et al. [38] found that improving the surface condition of samples and slowing water migration can help to inhibit the oxidation reaction process, thereby delaying the accumulation of lipid oxidation products. Combined with the results of this study, it can be concluded that the starch coating treatment may have slowed the rate of oxidative deterioration during refrigeration to some extent by improving the protective state of the fish fillet surface, reducing the opportunity for oxygen to contact the internal tissue, and delaying the lipid oxidation chain reaction. During long-term frozen storage (Figure 2D), the TBA values of both sample groups remained at low levels in the early stages, with relatively slow overall changes between 0 and 2 months, indicating that freezing conditions effectively suppressed lipid oxidation reactions during the initial storage period. As storage time increased, the TBA values of both groups gradually rose. In the control group, the rate of increase accelerated significantly after 4 months, particularly between 5 and 7 months, reaching 1.78 mg MDA/kg at 7 months. In contrast, the TBA value in the treatment group also showed a continuous upward trend, but the rate of increase remained lower than that of the control group. The difference between the two groups widened further in the later stages of storage. This indicates that under long-term storage conditions, the starch coating treatment still plays a positive role in delaying lipid oxidation. The lower TBA values in the treatment group may be attributed to the ability of the coating layer to reduce oxygen diffusion and surface dehydration, which are important factors promoting lipid oxidation in fish products. Compared with previous reports on edible coating preservation, the present results [39] show a similar trend in suppressing lipid oxidation. However, the increase in TBA during prolonged frozen storage also suggests that the protective effect of starch coating was limited and storage-time-dependent.

### 3.3. Spectral Analysis

**Figure 3:**
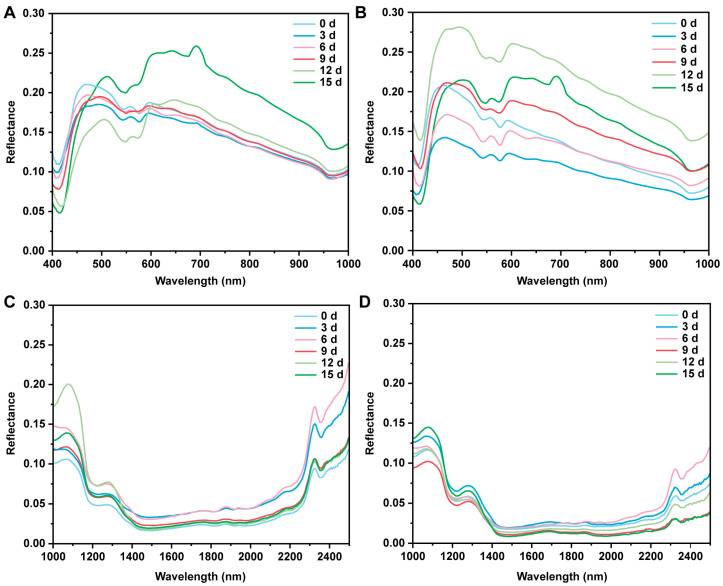
Mean reflectance spectra at different storage times during short-term storage. (**A**) Control group and (**B**) treatment group in the VIS. (**C**) Control group and (**D**) treatment group in the NIR.

**Figure 4:**
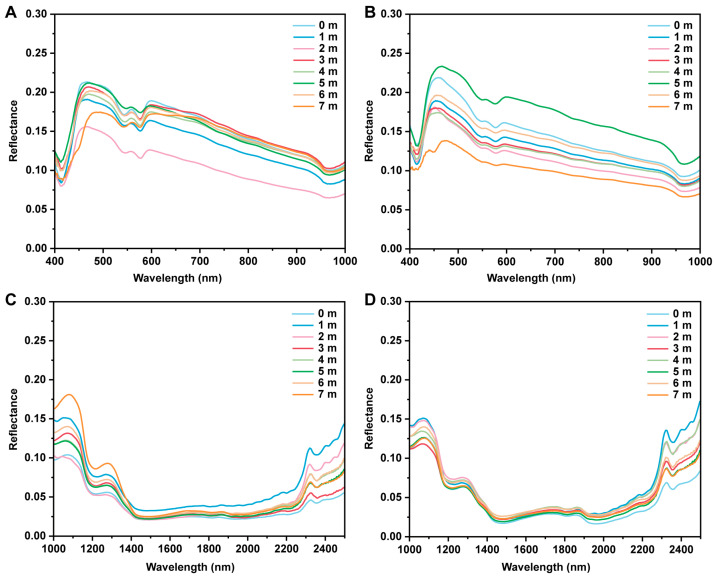
Mean reflectance spectra at different storage times during long-term storage. (**A**) Control group and (**B**) treatment group in the VIS. (**C**) Control group and (**D**) treatment group in the NIR.

Figure 3 and Figure 4 show the average VIS and NIR reflectance spectra of snakehead fish fillets at different storage times during short-term and long-term storage. The snakehead fish fillets in the control group and the treatment group maintained similar basic spectral profiles in both bands. However, as the refrigeration time increased, both the overall spectral positions and local absorption features underwent significant changes. In the VIS band (Figure 3A,B), differences between the two groups of samples were particularly evident in the 400–700 nm, with more pronounced fluctuations near 500–600 nm and 630 nm, indicating that the apparent color and pigment state of the samples continued to change during short-term refrigeration. These VIS changes may be associated with the transformation of myoglobin derivatives, pigment oxidation, and changes in surface scattering caused by tissue softening and water redistribution. The control group exhibited a more pronounced overall shift in reflectance toward higher wavelengths during the later stages of storage. The treatment group exhibited a relatively slower rate of change, indicating that the starch coating treatment mitigated alterations in the sample’s surface state and tissue scattering characteristics to some extent. Qin et al. [40] found that the absorption features in the 540–580 nm and approximately 630 nm regions are closely related to different forms of myoglobin. Hassoun et al. [41] suggested that tissue damage and changes in light scattering caused by storage or freeze–thaw cycles further enhance spectral differences. In the NIR band (Figure 3C,D), both groups of samples exhibited more pronounced temporal separation characteristics in the 1000–1400 nm and above-2200 nm regions, indicating that the internal water state, lipid oxidation levels, and protein structure of the samples all underwent continuous changes as storage progressed. The NIR response in these regions is mainly related to O-H, C-H, and N-H bond vibrations, which can reflect moisture migration, protein degradation, and lipid oxidation during storage. Compared to the VIS band, the NIR band is more sensitive to changes in internal composition. Furthermore, the control group exhibited a greater shift in spectral lines during the later stages of storage, while the changes in the treatment group were relatively gradual. These observations suggest that the control group showed more pronounced spectral changes related to internal composition and structural state during storage, whereas the treatment group showed relatively slower changes.

During long-term storage, snakehead fish fillets from both the control group and the treatment group exhibited good spectral distinguishability in the VIS and NIR bands. However, the patterns of change were more complex than those observed during short-term storage. In the VIS band (Figure 4A,B), distinct differences between the two groups of samples were still observable in 400–700 nm. The reflectance levels across different storage months did not exhibit a simple monotonic rise-and-fall relationship. Instead, significant fluctuations occurred at certain time points. This suggests that pigment oxidation, tissue structural rearrangement, and changes in surface scattering behavior may not occur synchronously under long-term storage conditions. Compared with short-term refrigerated storage, frozen storage may further introduce spectral complexity through ice crystal formation, thawing-related water redistribution, and changes in surface morphology. In contrast, the changes in the treatment group were relatively minor across certain months, suggesting that the starch coating treatment still provides a certain degree of buffering against changes in surface quality during long-term storage. In the NIR band (Figure 4C,D), both groups of samples exhibited a distinct separation trend in the regions around 1000–1200 nm, 1400 nm, and above 2300 nm, indicating that the redistribution of internal moisture, protein degradation, and the accumulation of lipid oxidation products continued to intensify as the duration of long-term storage increased. Cheng et al. [42] found that absorption features related to N-H, C=O, and O-H in the NIR region can effectively characterize changes in protein conformation and differences in moisture status. At the same time, the spectral line order across different months did not exhibit a simple linear trend but rather showed certain periodic fluctuations, indicating that the evolution of sample quality during long-term storage is influenced by multiple factors such as ice crystal formation, water migration, oxidative reactions, and changes in tissue structure, resulting in stronger nonlinear characteristics in the spectral response. Therefore, the spectral variations observed during long-term storage may reflect the combined effects of biochemical deterioration and physical structural changes, rather than a single quality factor. This result indicates that long-term storage samples exhibit more complex data distribution characteristics than short-term storage samples, which provides a spectral basis for the subsequent introduction of TL models to predict the freshness of long-term storage samples.

### 3.4. Freshness Prediction Based on Short-Term and Long-Term Spectral Data

The prediction performance of TVB-N and TBA contents in snakehead fish fillets based on short-term spectral data is summarized in Table 3. Overall, both VIS and NIR spectra showed the potential to predict freshness-related indicators, with *R*_T_^2^, *R*_CV_^2^, and *R*_P_^2^ values ranging from 0.71 to 0.87, 0.68 to 0.86, and 0.55 to 0.86, respectively. These results indicate that spectral information could capture freshness-related changes during short-term storage. Among all models, the VIS-based model for TBA prediction in the control group achieved the best testing performance, with an *R*_P_^2^ of 0.86 and an RMSE_P_ of 0.17, suggesting that VIS spectra were highly sensitive to lipid oxidation-related changes in fish fillets. Compared with NIR spectra, VIS spectra generally exhibited better testing set performance. For the control group, the VIS-based models achieved *R*_P_^2^ values of 0.81 and 0.86 for TVB-N and TBA contents, respectively, which were higher than those obtained using NIR spectra. Similarly, for the treatment group, VIS spectra also showed slightly better testing performance than NIR spectra for both indicators. The superior performance of VIS spectra may be attributed to the fact that short-term freshness changes are closely associated with surface color, tissue structure, oxidation state, and light scattering properties, which can be effectively reflected in the visible region. In contrast, NIR spectra showed acceptable training and validation performance, but their testing performance was relatively weak, indicating limited generalization ability on independent samples. This may be related to the relatively weak chemical changes in NIR-sensitive molecular bonds during short-term storage. Therefore, VIS spectra may provide more robust information for the rapid evaluation of early freshness changes in snakehead fish fillets.

**Figure 5:**
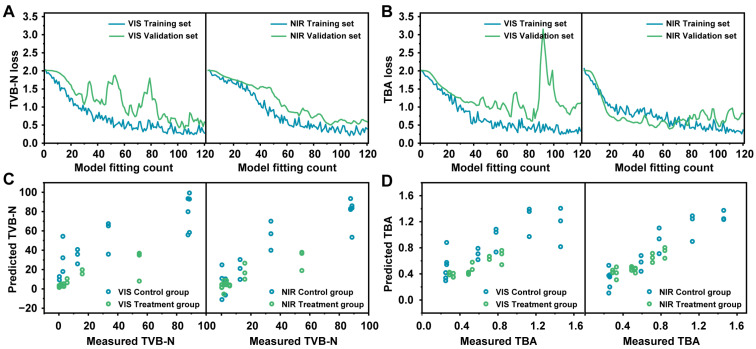
(**A**) Loss curve for predicting TVB-N content using the CNN model based on VIS and NIR spectra. (**B**) Loss curve for predicting TBA content using the CNN model based on VIS and NIR spectra. (**C**) Scatter plot of TVB-N content predicted by the CNN model based on VIS and NIR spectra. (**D**) Scatter plot of TBA content predicted by the CNN model based on VIS and NIR spectra. The above results are all based on the short-term domain data.

The training process and prediction performance of the CNN models based on short-term VIS and NIR spectral data are shown in Figure 5. The training losses for both TVB-N and TBA generally decreased with increasing fitting counts, indicating that the CNN models could extract freshness-related spectral features. However, the validation losses showed certain fluctuations, especially for the VIS-based TBA model, suggesting possible instability or overfitting during model training. This may be related to the limited number of samples, the nonlinear relationship between spectral responses and freshness indicators, and the sensitivity of deep learning models to sample distribution during training. The scatter plots further showed that TBA prediction exhibited a clearer relationship between measured and predicted values than TVB-N prediction, while TVB-N showed larger prediction dispersion, particularly at higher concentration levels. This difference may be because lipid oxidation-related spectral changes are more directly associated with surface colour and oxidation state, whereas TVB-N is affected by multiple spoilage-related processes and may show stronger sample heterogeneity. In addition, the treatment group generally showed lower measured TVB-N and TBA values than the control group, confirming that starch coating delayed freshness deterioration during short-term storage.

The prediction performance of TVB-N and TBA contents in snakehead fish fillets based on long-term spectral data is summarized in Table 4. Overall, both VIS and NIR spectra showed the ability to predict freshness-related indicators during long-term storage, with *R*_T_^2^, *R*_CV_^2^, and *R*_P_^2^ values ranging from 0.81 to 0.92, 0.70 to 0.86, and 0.62 to 0.86, respectively. These results indicate that long-term storage induced more pronounced biochemical and physical changes in fish fillets, which could be captured by spectral information. Among all models, the VIS-based model for TVB-N prediction in the treatment group achieved the best testing set performance, with an *R*_P_^2^ of 0.86 and RMSE_P_ of 8.36, suggesting that VIS spectra were highly sensitive to TVB-N-related freshness changes during long-term storage. Compared with NIR spectra, VIS spectra generally exhibited better prediction performance on the testing set. For the control group, the VIS-based models achieved *R*_P_^2^ values of 0.80 and 0.83 for TVB-N and TBA, respectively, which were higher than those obtained using NIR spectra. Similar trends were observed in the treatment group, especially for TVB-N prediction, where the VIS model achieved a higher *R*_P_^2^ and a lower RMSE_P_ than the NIR model. The superior performance of VIS spectra may be attributed to the fact that long-term storage causes obvious changes in surface color, tissue structure, oxidation state, and light scattering properties, which can be effectively reflected in the visible region. In contrast, NIR spectra showed acceptable training and validation performance, but their testing performance was relatively lower, indicating weaker generalization ability on independent samples. Therefore, VIS spectra may provide more robust information for evaluating long-term freshness changes in snakehead fish fillets.

**Figure 6:**
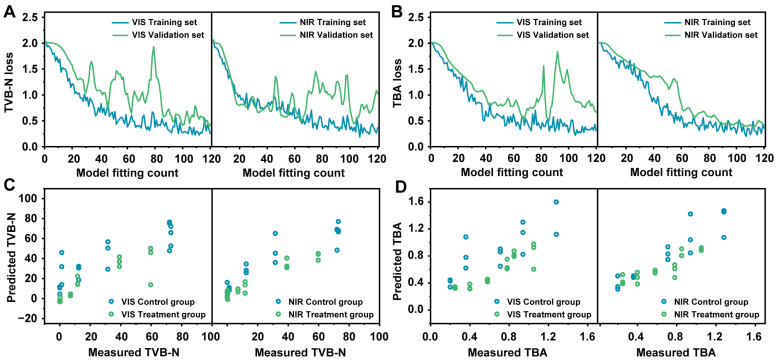
(**A**) Loss curve for predicting TVB-N content using the CNN model based on VIS and NIR spectra. (**B**) Loss curve for predicting TBA content using the CNN model based on VIS and NIR spectra. (**C**) Scatter plot of TVB-N content predicted by the CNN model based on VIS and NIR spectra. (**D**) Scatter plot of TBA content predicted by the CNN model based on VIS and NIR spectra. The above results are all based on the long-term domain data.

The training process and prediction performance of CNN models based on long-term VIS and NIR spectral data are shown in Figure 6. The training losses generally decreased with increasing fitting counts, indicating that the CNN models could extract freshness-related features from long-term storage spectra. However, the validation losses, especially for TVB-N prediction, showed noticeable fluctuations, suggesting that the spectral response of long-term samples was more complex and nonlinear. Compared with short-term storage, long-term frozen storage may introduce more variable spectral responses due to ice crystal formation, water redistribution, protein degradation, lipid oxidation, and structural changes, which can increase the difficulty of model generalization. The scatter plots showed positive relationships between measured and predicted values for both TVB-N and TBA, although larger dispersion was observed for TVB-N, particularly at high concentration levels. In comparison, TBA prediction exhibited a clearer trend, indicating that lipid oxidation-related changes could be effectively captured by spectral features. The relatively weaker performance of some NIR models may be associated with overlapping absorption bands and stronger matrix interference from water, protein, and lipid components, while VIS spectra may provide more stable information related to surface colour, oxidation, and tissue scattering. Moreover, the treatment group generally showed lower TVB-N and TBA levels than the control group did, confirming that starch coating delayed protein degradation and lipid oxidation during long-term storage.

### 3.5. Freshness Prediction Based on Transfer Learning

Table 5 summarizes the prediction performance of TVB-N and TBA contents in snakehead fish fillets based on the combination of short-term and long-term spectral data using the DT-CNN model. Overall, the DT-CNN model showed good predictive ability, with *R*_T_^2^, *R*_CV_^2^, and *R*_P_^2^ values ranging from 0.72 to 0.97, 0.66 to 0.90, and 0.66 to 0.88, respectively. These results indicate that the DT-CNN model could effectively integrate spectral information from different storage stages and improve the generalization ability of freshness prediction models. Among all models, the VIS-based model for TBA prediction in the control group achieved the best testing performance, with an *R*_P_^2^ of 0.88 and an RMSE_P_ of 0.19. In addition, the VIS-based model for TVB-N prediction in the treatment group also showed strong performance, with an *R*_P_^2^ of 0.85 and RMSE_P_ of 8.66. Compared with NIR spectra, VIS spectra consistently exhibited better testing set performance under the DT-CNN model. For the control group, the VIS-based models achieved *R*_P_^2^ values of 0.81 and 0.88 for TVB-N and TBA, respectively, which were higher than those of the NIR-based models. Similar trends were observed in the treatment group, where VIS spectra achieved higher *R*_P_^2^ values and lower RMSE_P_ values for both indicators. The superior performance of VIS spectra may be attributed to their sensitivity to surface color, tissue structure, oxidation state, and light scattering changes during fish storage. In contrast, although NIR spectra contain chemical information related to water, protein, and lipid changes, their testing performance was relatively lower, possibly due to overlapping absorption bands and stronger matrix interference. Compared with models established using short-term or long-term spectral data alone, the DT-CNN model showed improved or comparable testing set performance in most cases. For example, the VIS-Control-TBA model achieved a higher *R*_P_^2^ than the corresponding short-term and long-term models did, suggesting that the DT-CNN model enhanced the utilization of lipid oxidation-related spectral features across storage stages. Similarly, the DT-CNN model with NIR-control-TVB-N showed improved testing set performance, indicating that the DT-CNN model could partially alleviate the limited generalization ability of NIR models. However, the improvement was not consistent for all cases, as the DT-CNN model with NIR-treatment-TBA showed relatively weak testing set performance. Therefore, the DT-CNN model can improve the robustness of freshness prediction models, but the effectiveness is dependent on the spectral band, treatment condition, and freshness indicator.

**Figure 7:**
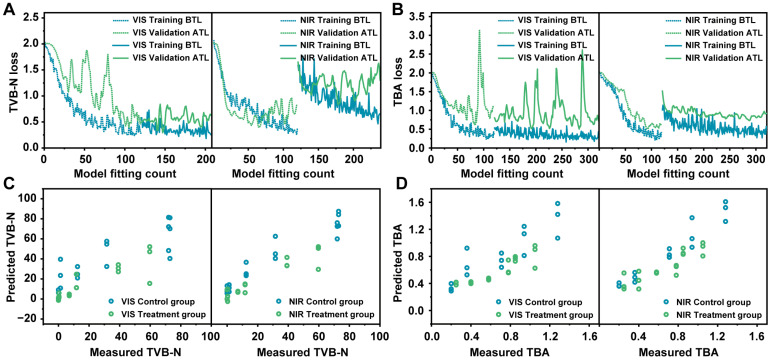
(**A**) Loss curve for predicting TVB-N content using the DT-CNN model based on VIS and NIR spectra. (**B**) Loss curve for predicting TBA content using the DT-CNN model based on VIS and NIR spectra. (**C**) Scatter plot of TVB-N content predicted by the DT-CNN model based on VIS and NIR spectra. (**D**) Scatter plot of TBA content predicted by the DT-CNN model based on VIS and NIR spectra. The above results are all based on the long-term domain data. BTL refers to training the DT-CNN model on short-term domain data prior to TL. ATL refers to training the DT-CNN model on long-term domain data following TL.

The training process and prediction performance of the DT-CNN model combined with TL for long-term domain samples are shown in Figure 7. Before TL, the loss values generally decreased, indicating that the model effectively learned spectral features from the short-term domain. After TL, the DT-CNN model further adapted the pre-trained model to the long-term domain, and the training losses remained relatively low. For TVB-N prediction, the VIS-based model showed more stable validation performance than the NIR-based model, suggesting that VIS spectra provided more robust information for long-term freshness prediction. For TBA prediction, both VIS and NIR models showed decreasing training losses, although fluctuations in validation loss indicated the nonlinear nature of lipid oxidation-related spectral responses. Although TL helped the model transfer general freshness-related spectral features from the short-term domain to the long-term domain, the distribution differences between refrigerated and frozen storage could not be completely eliminated. This may explain why the improvement in the DT-CNN model was not fully consistent across all indicators, spectral bands, and treatment groups. The scatter plots showed positive relationships between measured and predicted values for both TVB-N and TBA, confirming the feasibility of the DT-CNN model for long-term freshness prediction. Therefore, the DT-CNN model showed potential for improving prediction under limited long-term samples, but its performance was still affected by sample size, storage-domain differences, and the complexity of spectral-quality relationships. In addition, the treatment group generally exhibited lower TVB-N and TBA levels than the control group, further demonstrating the protective effect of starch coating against protein degradation and lipid oxidation.

## 4. Discussion

The deterioration of snakehead fish fillets during storage was reflected by the decrease in texture stability and the increase in pH, TVB-N, and TBA values, indicating progressive tissue softening, protein degradation, and lipid oxidation. Compared with the control group, the starch-coating treatment generally delayed the accumulation of products associated with spoilage and oxidation, suggesting a protective effect on freshness retention. The spectral changes in the VIS and NIR regions were consistent with these quality variations, with VIS spectra showing relatively better prediction performance, probably due to their sensitivity to surface colour, tissue structure, and oxidation-related changes. Moreover, the DT-CNN model improved or maintained predictive performance in most cases, demonstrating the feasibility of using short-term storage information to support freshness prediction under long-term storage conditions. It should be noted that this study focused on freshness assessment based on selected physicochemical indicators and hyperspectral responses, while tissue lipid content, moisture level, drip loss, and microbial counts were not directly measured. In addition, no formal subjective or expert sensory assessment was conducted; therefore, the fitness of fillets for consumption at later storage time points was not directly determined. TVB-N and TBA values were interpreted as freshness-related chemical indicators rather than direct criteria for edibility. Therefore, future studies should include these indicators to provide a more comprehensive evaluation of shelf-life and quality deterioration in starch-coated snakehead fish fillets.

## 5. Conclusions

This study developed a HSI method combined with TL for predicting the freshness of starch-coated snakehead fish fillets under short-term refrigerated and long-term frozen storage. The physicochemical results showed that storage promoted texture softening, pH increase, TVB-N accumulation, and lipid oxidation, whereas starch coating delayed freshness deterioration and improved storage stability. The prediction results showed that the DT-CNN model based on VIS spectra generally outperformed those of NIR spectra. After introducing TL, the DT-CNN model achieved improved or comparable performance in most cases, especially for prediction results of TBA and TVB-N with VIS spectra. Overall, HSI combined with TL provides a rapid, non-destructive, and robust strategy for monitoring the freshness of starch-coated snakehead fish fillets.

## Figures and Tables

**Table 1:** Sample distribution across different groups and domains.

	Days	0	3	6	9	12	15	-	-	Total
Short-term domain	CG	10	10	10	10	10	10	-	-	60
	TG	10	10	10	10	10	10	-	-	60
	Months	0	1	2	3	4	5	6	7	Total
Long-term domain	CG	8	8	8	8	8	8	6	6	60
	TG	8	8	8	8	8	8	6	6	60

Note: CG refers to the control group. TG refers to the treatment group.

**Table 2:** Changes in texture information and pH value of control and treatment groups during the short-term storage of snakehead fish fillets.

Group	Storage (d)	Hardness (g)	Elasticity	Cohesiveness	Adhesiveness (g)	Chewiness (g)	Resilience	pH
Control group	0	4420 ± 679 ^a^	0.154 ± 0.017 ^ab^	0.563 ± 0.019 ^cd^	2497 ± 450 ^a^	390 ± 53 ^a^	0.303 ± 0.018 ^a^	6.263 ± 0.015 ^d^
	3	4331 ± 595 ^a^	0.145 ± 0.015 ^ab^	0.532 ± 0.031 ^d^	2304 ± 382 ^a^	342 ± 49 ^ab^	0.285 ± 0.022 ^a^	6.170 ± 0.020 ^e^
	6	3875 ± 485 ^ab^	0.130 ± 0.019 ^b^	0.516 ± 0.023 ^d^	2243 ± 403 ^a^	295 ± 56 ^ab^	0.253 ± 0.008 ^b^	6.293 ± 0.006 ^d^
	9	3246 ± 273 ^bc^	0.158 ± 0.007 ^a^	0.605 ± 0.057 ^bc^	1826 ± 220 ^a^	287 ± 43 ^b^	0.214 ± 0.006 ^c^	6.510 ± 0.020 ^c^
	12	2903 ± 464 ^c^	0.156 ± 0.007 ^ab^	0.669 ± 0.043 ^ab^	2247 ± 304 ^a^	305 ± 45 ^ab^	0.206 ± 0.010 ^c^	7.090 ± 0.030 ^b^
	15	2546 ± 384 ^c^	0.152 ± 0.010 ^ab^	0.692 ± 0.045 ^a^	2359 ± 354 ^a^	320 ± 51 ^ab^	0.194 ± 0.013 ^c^	7.293 ± 0.038 ^a^
Treatment group	0	3131 ± 652 ^a^	0.199 ± 0.016 ^a^	0.702 ± 0.018 ^a^	2201 ± 368 ^a^	425 ± 68 ^a^	0.343 ± 0.019 ^a^	6.333 ± 0.021 ^c^
	3	2864 ± 447 ^ab^	0.198 ± 0.025 ^a^	0.562 ± 0.082 ^b^	1239 ± 402 ^b^	276 ± 46 ^b^	0.266 ± 0.011 ^b^	6.307 ± 0.021 ^d^
	6	2651 ± 437 ^ab^	0.195 ± 0.038 ^a^	0.547 ± 0.077 ^b^	985 ± 223 ^b^	192 ± 25 ^c^	0.264 ± 0.024 ^b^	6.210 ± 0.020 ^c^
	9	2544 ± 373 ^abc^	0.193 ± 0.017 ^a^	0.587 ± 0.053 ^ab^	1479 ± 393 ^ab^	274 ± 28 ^b^	0.211 ± 0.007 ^c^	6.320 ± 0.020 ^c^
	12	2042 ± 279 ^bc^	0.182 ± 0.019 ^a^	0.590 ± 0.084 ^ab^	1548 ± 415 ^ab^	281 ± 38 ^b^	0.208 ± 0.019 ^c^	6.740 ± 0.020 ^b^
	15	1742 ± 384 ^c^	0.165 ± 0.021 ^a^	0.664 ± 0.067 ^ab^	1578 ± 431 ^ab^	289 ± 45 ^b^	0.215 ± 0.012 ^c^	6.923 ± 0.015 ^a^

Note: Data are presented as mean ± standard deviation (the number of replicate samples is 6). Different lowercase letters within the same column indicate significant differences (*p* < 0.05).

**Table 3:** Freshness prediction results of snakehead fish fillets based on short-term spectral data.

Band	Group	Indicator	Training Set		Validation Set		Testing Set	
			*R*_T_^2^	RMSE_T_	*R*_CV_^2^	RMSE_CV_	*R*_P_^2^	RMSE_P_
VIS	Control	TVB-N	0.82	15.71	0.71	20.10	0.81	16.11
		TBA	0.81	0.19	0.68	0.25	0.86	0.17
	Treatment	TVB-N	0.87	6.79	0.71	10.32	0.66	11.04
		TBA	0.87	0.07	0.79	0.09	0.72	0.10
NIR	Control	TVB-N	0.83	15.39	0.75	18.76	0.61	23.34
		TBA	0.87	0.16	0.86	0.17	0.59	0.28
	Treatment	TVB-N	0.71	10.20	0.80	8.62	0.55	12.83
		TBA	0.75	0.10	0.81	0.08	0.70	0.11

**Table 4:** Freshness prediction results of snakehead fish fillets based on long-term spectral data.

Band	Group	Indicator	Training Set		Validation Set		Testing Set	
			*R*_T_^2^	RMSE_T_	*R*_CV_^2^	RMSE_CV_	*R*_P_^2^	RMSE_P_
VIS	Control	TVB-N	0.84	12.36	0.73	15.83	0.80	13.78
		TBA	0.83	0.22	0.75	0.27	0.83	0.22
	Treatment	TVB-N	0.92	6.25	0.84	8.99	0.86	8.36
		TBA	0.85	0.11	0.78	0.13	0.73	0.14
NIR	Control	TVB-N	0.84	12.12	0.77	14.69	0.63	18.52
		TBA	0.87	0.20	0.84	0.21	0.62	0.33
	Treatment	TVB-N	0.89	7.29	0.86	8.30	0.70	12.18
		TBA	0.81	0.12	0.70	0.15	0.71	0.15

**Table 5:** Freshness prediction results of snakehead fish fillets based on short-term and long-term spectral data combined with the DT-CNN model.

Band	Group	Indicator	Training Set		Validation Set		Testing Set	
			*R*_T_^2^	RMSE_T_	*R*_CV_^2^	RMSE_CV_	*R*_P_^2^	RMSE_P_
VIS	Control	TVB-N	0.82	12.99	0.74	15.71	0.81	13.29
		TBA	0.84	0.21	0.78	0.25	0.88	0.19
	Treatment	TVB-N	0.97	4.05	0.80	9.88	0.85	8.66
		TBA	0.93	0.07	0.76	0.13	0.76	0.13
NIR	Control	TVB-N	0.88	10.52	0.79	13.98	0.66	17.91
		TBA	0.92	0.16	0.90	0.17	0.66	0.31
	Treatment	TVB-N	0.85	8.56	0.87	8.02	0.70	12.12
		TBA	0.72	0.14	0.66	0.16	0.67	0.16

## Data Availability

The data presented in this study are available on request from the corresponding author due to institutional data-management requirements and research ethics restrictions.
